# Supplementary material for: Human NMD ensues independently of stable ribosome stalling
Source: Nat Commun. 2020 Aug 17;11:4134. doi: 10.1038/s41467-020-17974-z (PMC7431590; doi:10.1038/s41467-020-17974-z)
Supplement: Supplementary file 1 — Supplementary Information [file 41467_2020_17974_MOESM1_ESM.pdf]

Supplementary information

Human NMD ensues independently of stable ribosome stalling

Evangelos D. Karousis, Lukas-Adrian Gurzeler, Giuditta Annibaldis, René Dreos and Oliver Mühlemann

Supplementary Figure 1: Optimization of *in vitro* translation conditions

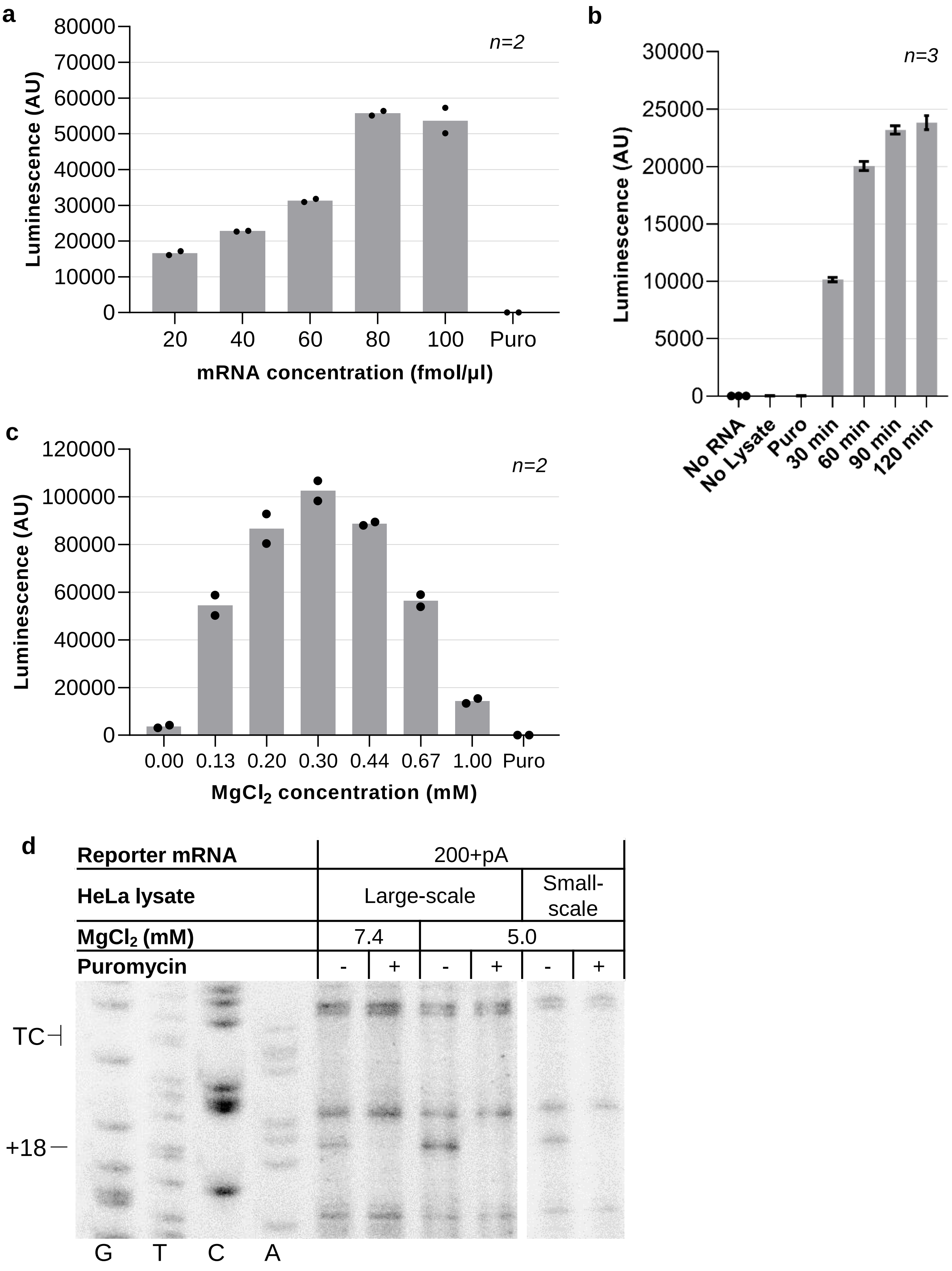

a. *In vitro* translation using different concentrations of 200+pA mRNA. Rluc activity measurements are depicted as arbitrary units (AU) of luminescence. The dots depict the values of two individual experiments. **b.** Time-course of 200+pA in vitro translation reaction using 40 fmol/µL. Rluc activity measurements are depicted as in (a), mean values and SD of three indepent experiments are shown. **c.** Mg^{2+} titration for in vitro translation reactions using 40 fmol/µl 200+pA mRNA as substrate. Rluc activity measurements are depicted as in (a). **d.** Toeprint analysis with reporter mRNA containing a 200 nts long 3´UTR and an 80 nts long poly(A) tail (200+pA). *In vitro* translation was performed in large scale (from > 10^8 cells, yielding > 500 µl lysate) or small-scale lysates (from < $4x10^7$ cells, yielding less than 200 µl lysate). Translation-competent HeLa lysates were incubated for 50 min in the presence or absence of puromycin and the toeprint reactions were performed under different $MgCl_2$ concentrations. Sanger sequencing reactions were run in parallel (G, T, C, A). The positions of termination codon (TC) and the toeprint band 18 nucleotides downstream of the first nucleotide of the TC (+18), corresponding to ribosomes located at the TC, are indicated. Source data are provided as a Source Data File.

Supplementary Figure 2: Assessment of degradation in the context of toeprint assays

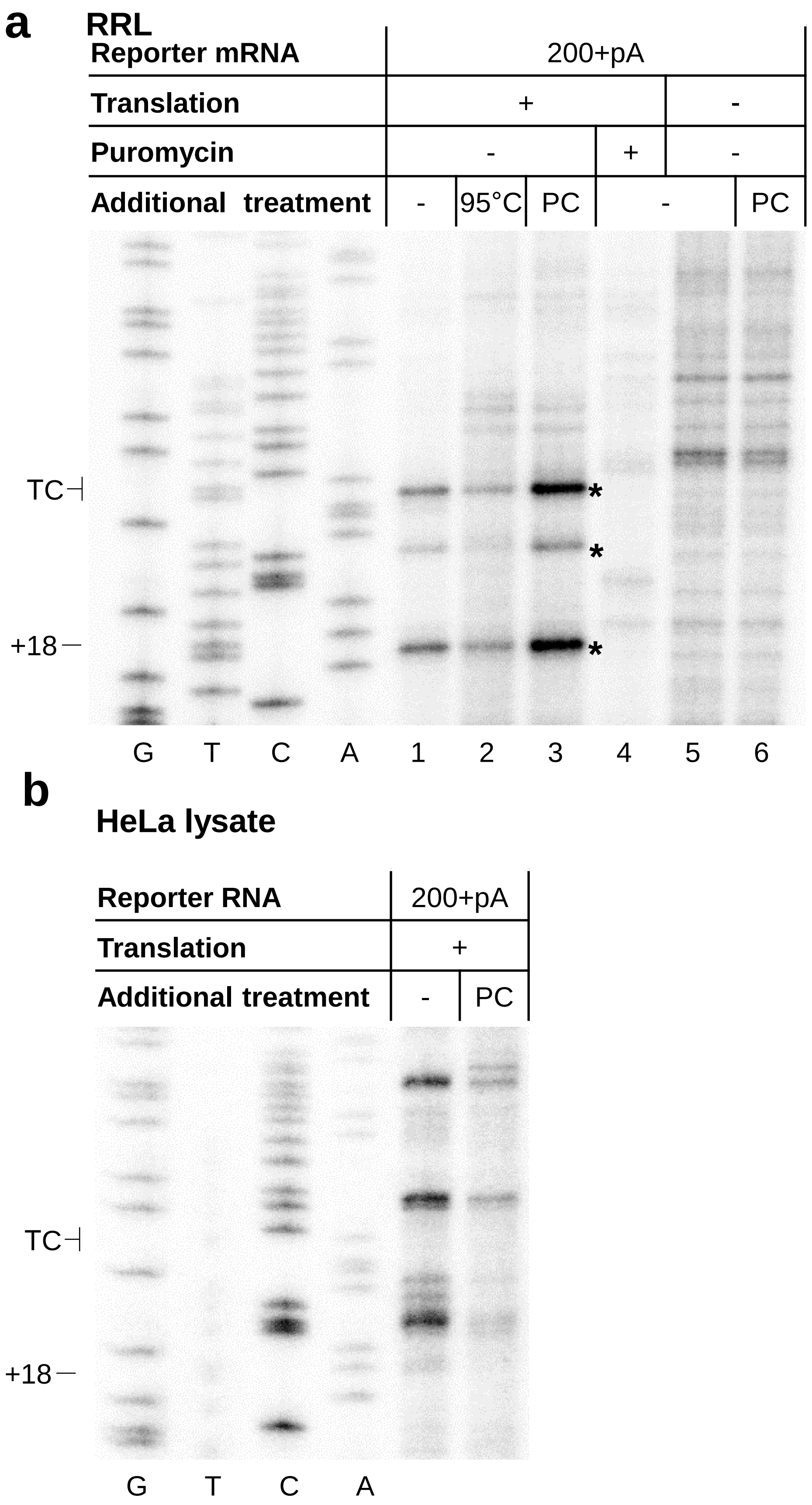

Supplementary Figure 2

a. Toeprint analysis with the 200+pA reporter RNA translated in rabbit reticulocyte lysate (RRL) for 30 min in the presence or absence of puromycin. The toeprint reaction was performed after either incubation at 95°C to denature proteins and ribosomal complexes (95°C) or by phenol-chloroform extraction (PC). To visualize co-translational degradation products, untranslated RNA was extracted in parallel. Sanger sequencing reactions were run in parallel (G, T, C, A) to locate the positions of the signals. The position of the TC and the toeprint band corresponding to the ribosomes at the TC (+18) are indicated. Translation-dependent cleavage products are depicted by asterisks.

b. Toeprint analysis with the 200+pA reporter mRNA translated in translation-competent HeLa lysates for 50 min. Before the reverse transcription step, phenol-chloroform extraction (PC) was performed to identify potential co-translational RNA cleavage events. Sanger sequencing reactions were run in parallel (G, T, C, A) to locate the positions of the toeprints. The position of the TC and the toeprint band corresponding to the ribosomes at the TC (+18) are indicated. Source data are provided as a Source Data File.

Supplementary Figure 3

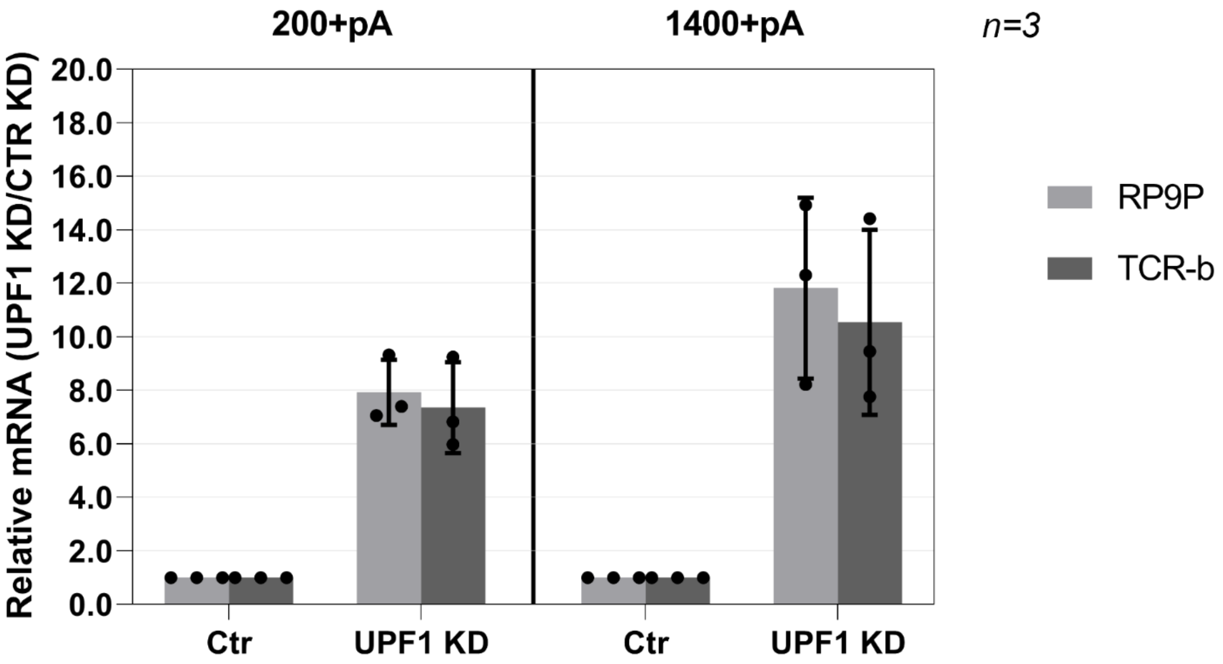

Relative levels of a TCR-β NMD reporter mRNA and the endogenous NMD-sensitive Retinitis Pigmentosa 9 Pseudogene (RP9P) mRNA measured by RT-qPCR from cells depleted for UPF1 (UPF1 KD) or with a control knockdown (CTRL KD) and expressing either of the Rluc reporter genes 200+pA or 1400+pA. Mean values ± standard deviations of 3 biological replicates are shown, values of the individual experiments are indicated by dots.

List of primers and RNA oligonucleotides

**Ampicillin resistance gene amplification**	5’-AAT TTC TAG AAT TGT TGC CGG GAA GCT AGA GTA AG-3’
	5‘-AAT TTC TAG ATG AGT ATT CAA CAT TTC CGT GTC G-3‘
**Site directed mutagenesis Reporter A**	5´-CAA ATG TGG TAT GGC TGA TTA GAT CCT CTA GAA TTC CTG CTC-3´
**Site directed mutagenesis Reporter B**	5´-CAA ATG TGG TAT GGC TGA TTA GAT CCT CAA GAA TTC CTG CTC-3´
**Site directed mutagenesis Reporter C**	5´-AAA TGT GGT ATG GCT GAT TGG ATC CTC AAG AAT TCC TGC-3´
**RLuc ORF amplification**	5´-GGG CCC ATG GCT TCC AAG GTG TAC GA-3´ and 5´-GGT ACC AAC AAC AAC AAT TGC ATT CA-3´
	5´-GGT ACC AAC AAC AAC AAT TGC ATT CA-3´
**Site directed mutagenesis Reporter Kozak sequence**	5´-CTG GCT AGC GTT TAA ACG CCA CCA TGG CTT CCA AGG TGT-3´
**Fusion PCR for p1400**	Fragment 1:<br>5´-GGG CCC ATG GCT TCC AAG GTG TAC GA-3´ and 5´-TGG CGA TGA GAA CAA CAA CAA TTG CAT TCA-3´
	Fragment 2:<br>5´-TGT TGT TGT TCT CAT CGC CAA TTG TTG CC-3´ and 5´-GGT ACC CTA GAT GAG TAT TC-3´
**Toeprint primer**	5’-TCA GGT TCA GGG GGA GGT G-3’
**UPF1 siRNA**	5’-GAUGCAGUUCCGCUCCAUU-3’
**ABCE1 siRNA**	5´-GAG GAG AGU UGC AGA GAU UU dTdT-3´
**Negative CTR siRNA**	5´-AGG UAG UGU AAU CGC CUU G dTdT-3´
**beta-actin qPCR assay**	5´-TCC ATC ATG AAG TGT GAC GT-3´
	5´-TAC TCC TGC TTG CTG ATC CAC-3´
**Mini-TCRβ reporter qPCR assay**	5´-AGT TGG CTT CCC TTT CTC AG-3´
	5´-CTT GGG TGG AGT CAC ATT TC-3´
**Retinitis Pigmentosa 9 Pseudogene (RP9P) qPCR assay**	5´-CAA GCG CCT GGA GTC CTT AA-3´
	5´-AGG AGG TTT TTC ATA ACT CGT GAT CT-3´
**humanized Renilla luciferase qPCR assay**	5´-CCC CGA GCA ACG CAA AC-3´
	5´-GCA CGT TCA TTT GCT TGC A-3´

Supplementary Table 1
