# Supplementary material for: Human NMD ensues independently of stable ribosome stalling
Source: Nat Commun. 2020 Aug 17;11:4134. doi: 10.1038/s41467-020-17974-z (PMC7431590; doi:10.1038/s41467-020-17974-z)
Supplement: Supplementary file 3 — Reporting Summary [file 41467_2020_17974_MOESM3_ESM.pdf]

nature research

Corresponding author(s): Oliver Mühlemann

Last updated by author(s): Jul 7, 2020

Reporting Summary

Nature Research wishes to improve the reproducibility of the work that we publish. This form provides structure for consistency and transparency in reporting. For further information on Nature Research policies, see Authors & Referees and the Editorial Policy Checklist.

Statistics

For all statistical analyses, confirm that the following items are present in the figure legend, table legend, main text, or Methods section.

n/a	Confirmed	
☐	☒	The exact sample size (n) for each experimental group/condition, given as a discrete number and unit of measurement
☐	☒	A statement on whether measurements were taken from distinct samples or whether the same sample was measured repeatedly
☐	☒	The statistical test(s) used AND whether they are one- or two-sided<br>*Only common tests should be described solely by name; describe more complex techniques in the Methods section.*
☒	☐	A description of all covariates tested
☒	☐	A description of any assumptions or corrections, such as tests of normality and adjustment for multiple comparisons
☐	☒	A full description of the statistical parameters including central tendency (e.g. means) or other basic estimates (e.g. regression coefficient) AND variation (e.g. standard deviation) or associated estimates of uncertainty (e.g. confidence intervals)
☐	☒	For null hypothesis testing, the test statistic (e.g. F, t, r) with confidence intervals, effect sizes, degrees of freedom and P value noted<br>*Give P values as exact values whenever suitable.*
☒	☐	For Bayesian analysis, information on the choice of priors and Markov chain Monte Carlo settings
☒	☐	For hierarchical and complex designs, identification of the appropriate level for tests and full reporting of outcomes
☒	☐	Estimates of effect sizes (e.g. Cohen's d, Pearson's r), indicating how they were calculated

Our web collection on statistics for biologists contains articles on many of the points above.

Software and code

Policy information about availability of computer code

Data collection	No software was used for data collection
Data analysis	ImageJ 1.52p and Rotor-Gene 6200 version 2.3.1 software

For manuscripts utilizing custom algorithms or software that are central to the research but not yet described in published literature, software must be made available to editors/reviewers. We strongly encourage code deposition in a community repository (e.g. GitHub). See the Nature Research guidelines for submitting code & software for further information.

Data

Policy information about availability of data

All manuscripts must include a data availability statement. This statement should provide the following information, where applicable:

- Accession codes, unique identifiers, or web links for publicly available datasets
- A list of figures that have associated raw data
- A description of any restrictions on data availability

Ribo-Seq data used in Figure 5 has been deposited in the Gene Expression Ominibus (GEO) under accesion numbers:
GSM4256659 SK1_M (L6)
GSM4256660 SK2_M (L6)
GSM4256661 SK3_M (L6)
GSM4256665 SK1_M (L7)
GSM4256666 SK2_M (L7)
GSM4256667 SK3_M (L7)
Each biological replicate (SK1-3) has been sequenced in two lanes (L6 and L7) and deposited individually. Total RNA sequencing data for UPF1 KD conditions that were used to compile a list of NMD-sensitive transcripts are deposited under accession numbers: GSM4407914, GSM4407915, GSM4407916. The data will be made public upon acceptance of the manuscript. Meanwhile, for review purpose only, the data can be accessed with the following token: ozyjskeknjidnsz

Field-specific reporting

Please select the one below that is the best fit for your research. If you are not sure, read the appropriate sections before making your selection.

☒ Life sciences ☐ Behavioural & social sciences ☐ Ecological, evolutionary & environmental sciences

For a reference copy of the document with all sections, see nature.com/documents/nr-reporting-summary-flat.pdf

Life sciences study design

All studies must disclose on these points even when the disclosure is negative.

Sample size	A total of minimum three biological measurements has been selected following common practice for molecular biology experiments.
Data exclusions	No data are excluded, for toeprint assays, one representative replicate is shown.
Replication	Three biological replicates were performed for all experiments performed in HeLa cell lines. In vitro translation experiments and toeprint assays were performed at least three times and were considered as valid when the same result was observed in all replicates with valid controls. All attempts at replication were successful
Randomization	Not applicable. We did not perform experiments that require randomization.
Blinding	Not applicable. We did not perform experiments that require randomization.

Reporting for specific materials, systems and methods

We require information from authors about some types of materials, experimental systems and methods used in many studies. Here, indicate whether each material, system or method listed is relevant to your study. If you are not sure if a list item applies to your research, read the appropriate section before selecting a response.

Materials & experimental systems

n/a	Involved in the study	
☐	☒	Antibodies
☐	☒	Eukaryotic cell lines
☒	☐	Palaeontology
☒	☐	Animals and other organisms
☒	☐	Human research participants
☒	☐	Clinical data

Methods

n/a	Involved in the study	
☒	☐	ChIP-seq
☒	☐	Flow cytometry
☒	☐	MRI-based neuroimaging

Antibodies

Antibodies used	UPF1 (Bethyl A300-038A, 1:1000), ABCE1 (Abcam, ab185548, 1:1000), beta-actin (Sigma Aldrich A5060, 1:2000), Rluc (Thermo Fisher PA5-32210, 1:600), Tyr-Tubulin (Sigma T9028, 1:5000).
Validation	Validation of antibodies is done by western blot in the corresponding manufacturer's websites and for UPF1 and ABCE1 after siRNA-mediated knockdowns in the present study.

Eukaryotic cell lines

Policy information about cell lines

Cell line source(s)	HeLa (ATCC; CCL2), HeLa TCRβ PTC 68 (Hela tetR clone 9) was developed and validated in our lab (Eberle A et al., PLoS Biol. 6, e92 (2008).)
Authentication	HeLa TCRβ PTC 68 (Hela tetR clone 9) was authenticated by qPCR by readout of the TCRβ constructs (Eberle A et al., PLoS Biol. 6, e92 (2008).)
Mycoplasma contamination	All cell lines were tested negative for Mycoplasma contamination

Commonly misidentified lines<br>(See ICLAC register)	No commonly misidentified cell lines were used in the study.
